# Investigating a new neuromodulation treatment for brain disorders using synchronized activation of multimodal pathways

**DOI:** 10.1038/srep09462

**Published:** 2015-03-25

**Authors:** Craig D. Markovitz, Benjamin T. Smith, Cory D. Gloeckner, Hubert H. Lim

**Affiliations:** 1 Department of Biomedical Engineering, University of Minnesota, Minneapolis, MN USA; 2 Department of Otolaryngology-Head and Neck Surgery, University of Minnesota, Minneapolis, MN USA; 3 Institute for Translational Neuroscience, University of Minnesota, Minneapolis, MN USA

## Abstract

Neuromodulation is an increasingly accepted treatment for neurological and psychiatric disorders but is limited by its invasiveness or its inability to target deep brain structures using noninvasive techniques. We propose a new concept called Multimodal Synchronization Therapy (mSync) for achieving targeted activation of the brain via noninvasive and precisely timed activation of auditory, visual, somatosensory, motor, cognitive, and limbic pathways. In this initial study in guinea pigs, we investigated mSync using combined activation of just the auditory and somatosensory pathways, which induced differential and timing dependent plasticity in neural firing within deep brain and cortical regions of the auditory system. Furthermore, by varying the location of somatosensory stimulation across the body, we increased or decreased spiking activity across different neurons. These encouraging results demonstrate the feasibility of systematically modulating the brain using mSync. Considering that hearing disorders such as tinnitus and hyperacusis have been linked to abnormal and hyperactive firing patterns within the auditory system, these results open up the possibility for using mSync to decrease this pathological activity by varying stimulation parameters. Incorporating multiple types of pathways beyond just auditory and somatosensory inputs and using other activation patterns may enable treatment of various brain disorders.

Neuromodulation is rapidly growing as a treatment option for various brain disorders^1^. Clinical outcomes for invasive approaches, including deep brain or cortical stimulation, have been encouraging for some neurological conditions such as Parkinson's tremors and tinnitus^2^,^3^,^4^,^5^. However, they are used only in a limited patient population due to their surgical risks, high cost, and need for extensive fitting in specialized clinics^2^,^3^,^6^,^7^. Noninvasive stimulation techniques, including transcranial magnetic stimulation and transcranial direct current stimulation, have also shown efficacy in treating some patients with brain conditions such as depression, obsessive compulsive disorder, and chronic pain^8^,^9^. However, these methods generally produce broad neuromodulatory effects across the cortex and cannot target deeper structures without causing extensive cortical activation^1^. We propose an alternative approach for activating deep brain and cortical regions in a noninvasive way, which we call Multimodal Synchronization Therapy (**mSync**). mSync takes advantage of the dense and topographic interconnectivity of the nervous system in which the brain integrates information cortically and subcortically across auditory, visual, somatosensory, motor, cognitive, and limbic pathways^10^,^11^,^12^. By combining stimulation across these modalities at precise timing intervals, we propose the ability to achieve targeted activation of specific populations of neurons while diffusely and weakly activating other neural populations, based on the assumption that different neurons have varying combinations and timing of these inputs.

Several examples within the research literature have already shown the clinical potential of using multimodal integration to treat various brain disorders. Mirror therapy, which provides visual feedback for amputees while performing a motor task associated with their missing limb, can inhibit phantom limb pain^11^,^13^. Stimulation of the trigeminal nerve, which is associated with somatosensory and motor pathways, has shown some success for treating epilepsy and depression^14^,^15^. Visual cueing and auditory startle techniques can initiate movement in Parkinson's patients with freezing symptoms^16^. Facial, gaze, jaw, and neck movements or somatosensation can modulate the tinnitus percept in a subset of patients^17^,^18^. These studies demonstrate the brain's immense capacity to integrate multimodal inputs, which may be utilized to alter pathogenic neural activity. We propose using mSync with systematically chosen parameters to activate multiple pathways in a more temporally precise manner than the clinical cases described above in order to treat different brain conditions.

The concept and success of mSync is based on four assumptions: (1) the aberrant neural populations driving an abnormal brain condition are able to be activated or modulated by multiple inputs/pathways; (2) some or all of these pathways can be activated noninvasively; (3) using appropriate timing of activation, these pathways will elicit converging synchronized activation of the targeted neural population while eliciting temporally diffuse activation of other populations due to differences in latencies of convergence; and (4) through repetition, the converging activation can induce long-lasting neural plasticity relevant for treating the abnormal brain state.

Before investigating and testing each of these assumptions, we first needed to assess whether mSync could even modulate the brain in a systematic manner that would be relevant for treating a brain disorder. For this initial study, we investigated the mSync concept in relation to tinnitus and hyperacusis, which are hearing disorders that affect the quality of life of more than 5% of the population^19^,^20^,^21^,^22^ (statistics also from Centers for Disease Control and Prevention). Unfortunately, there are no reliable treatments for these disorders.

Since tinnitus and hyperacusis have been linked to hyperactivity and other abnormal brain patterns throughout the central auditory system^19^,^20^,^21^,^23^, we recorded neural activity in two key regions along the auditory pathway: the inferior colliculus (**IC**), a major subcortical auditory center located in the midbrain, and the auditory cortex (**AC**), a region associated with sound perception. We compared changes in auditory activity induced by several mSync paradigms to those induced by control paradigms. For this study, the parameters were confined to paired stimulation of just auditory and somatosensory pathways to simplify the experimental protocol, focusing on the neuromodulatory effects caused by different interstimulus delays and stimulation locations across the body.

## Results

### Experiment 1

**Figure 1:**
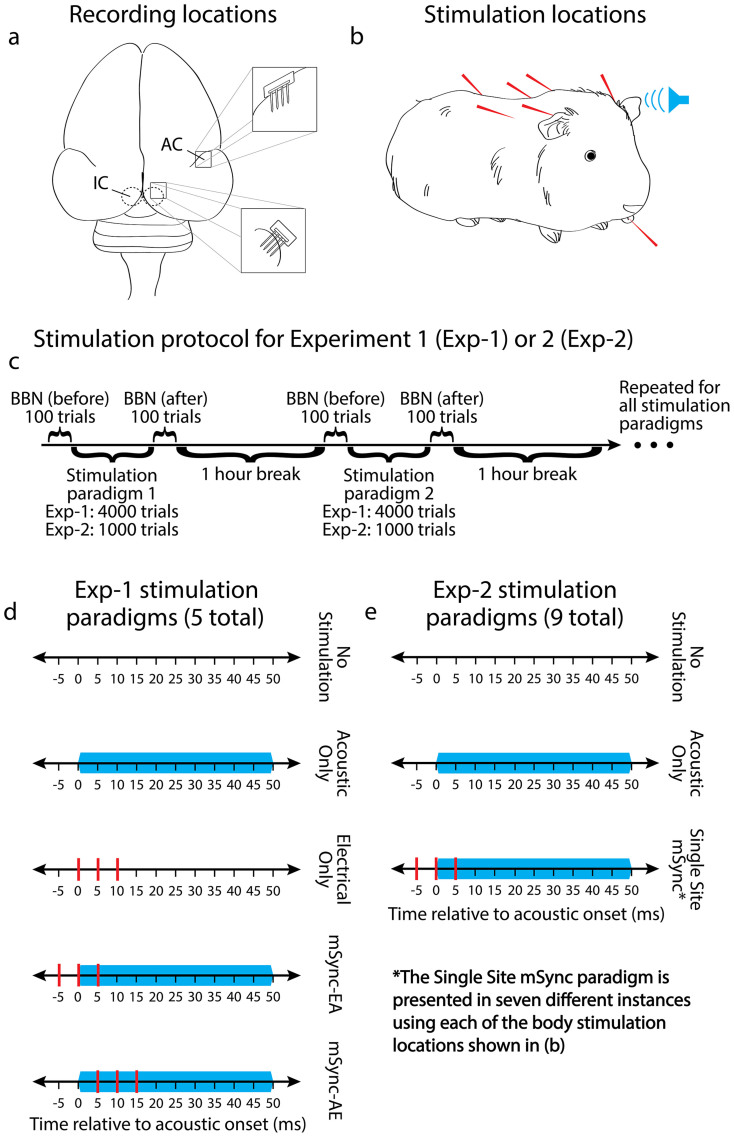
Experimental protocol. (a) Neural recordings were made with 32-site NeuroNexus arrays in the right inferior colliculus (IC; its central nucleus) and auditory cortex (AC; its primary area). (b) Broadband acoustic stimulation was presented via a speaker coupled to the animal's left ear canal, and somatosensory electrical stimulation was presented via subcutaneous needle electrodes placed onto or within the tongue, neck, left mastoid, and right mastoid for Experiment 1 and onto or within the tongue, neck, left mastoid, right mastoid, left shoulder, right shoulder, and back for Experiment 2. (c) The stimulation protocol for Experiments 1 and 2 consisted of comparing the neural activity within the IC and AC in response to 100 trials of 70 dB SPL broadband noise (BBN) presented before and after a given stimulation paradigm, which are shown in (d) and (e) for Experiments 1 and 2, respectively. The stimulation paradigm order was randomized across animals with a one hour break between paradigms to reduce cumulative effects. (d) Single trials (inter-trial interval of 500 ms) are shown for the control (top three) and experimental (bottom two) paradigms used in Experiment 1. The blue bars represent a 50 ms duration (0.5 ms rise/fall time), 50 dB SPL BBN presented to the animal's left ear and the red lines are electrical stimulation pulses presented to the different body locations. Analysis was performed on 100 trials of acoustic-driven activity in response to 70 dB BBN recorded before and after 4,000 consecutive trials of each paradigm. For the Electrical Only, mSync-EA, and mSync-AE paradigms, each body location was stimulated for 1,000 trials in a randomized order across the four body locations for a total of 4,000 trials. (e) Single trials are shown for the control (top two) and experimental (bottom, Single Site mSync) paradigms used in Experiment 2. Each paradigm consisted of 1,000 consecutive trials of stimulation. Note that Single Site mSync is the same as mSync-EA from Experiment 1 except it consisted of only one body site at a time for all 1,000 trials. The seven different body locations shown in (b) were used, resulting in seven different experimental paradigms.

Penetrating 32-site electrode arrays were implanted into primary auditory cortex (**A1**) and the central nucleus of the IC (**ICC**) of ketamine-anesthetized guinea pigs (13 animals) for recording acoustic-driven responses (Figure 1a). We performed experiments in anesthetized animals with normal hearing to simplify the preparation while still being able to achieve the goals of our study. Success with these experiments would then justify and guide later studies in awake and disease-specific animal models to further expand upon the results found under anesthesia. Subcutaneous stimulation needles were placed onto or within the tongue, neck, left mastoid, and right mastoid for somatosensory stimulation, and a speaker was coupled to the left ear canal for presentation of broadband noise (Figure 1b). In treating human patients, mSync will be implemented noninvasively. However, for ensuring consistent placement and a reliable conductive interface for somatosensory stimulation in animals, subcutaneous needle electrodes were used for this initial study. Noninvasive stimulation, including the use of current, pressure, ultrasound, and thermal modalities, will be investigated in future experiments based on the findings from this study. In terms of neural recording, we identified the electrode sites (out of the 32 total sites per brain region and animal) that were within the ICC or A1 based on well-established acoustic-driven properties characteristic of these brain regions. Further details on the selection and consistent placement of the stimulation and recording electrodes are provided in the Methods.

Five stimulation paradigms were presented: two mSync paradigms (**mSync-EA** with electrical body stimulation leading acoustic stimulation by 5 ms and **mSync-AE** with acoustic stimulation leading electrical body stimulation by 5 ms) and three controls (No Stimulation, Acoustic Only, and Electrical Only), as shown in Figure 1d. For the conditions using body stimulation, we did not initially know which locations would be effective in inducing auditory plasticity. Based on the clinical cases in modulating tinnitus with manipulations of the head or neck described in the Introduction^17^,^18^, we initially selected the neck, tongue, left mastoid, and right mastoid for stimulation. For each stimulation paradigm, we stimulated all four body sites in a random sequence to focus our study on the timing between somatosensory and acoustic stimulation rather than any one specific body location. Then in Experiment 2, we investigated the effects of individual body locations. Acoustic-driven responses to 70 dB SPL broadband noise were compared immediately before and after 4,000 trials (at 2 trials/s) of each stimulation paradigm (Figure 1c) using an unequal variance two-tailed t-test on ranked data (i.e., spike counts) across trials with significance defined as p < 0.01^24^, which is further explained in the Methods. Since this study is our first set of experiments investigating the feasibility of mSync, we initially analyzed the changes in acoustic-driven activity because they can be reliably recorded and compared for the different paradigms in an anesthetized preparation and using multi-unit recordings. Future studies will investigate other neural patterns linked to tinnitus or hyperacusis, such as spontaneous activity, synchrony, and temporal firing^19^,^20^,^23^, which can be more reliably characterized for data obtained in awake preparations and/or using single-unit recordings.

### Experiment 1: mSync paradigms alter acoustic-driven firing of ICC and A1

**Figure 2:**
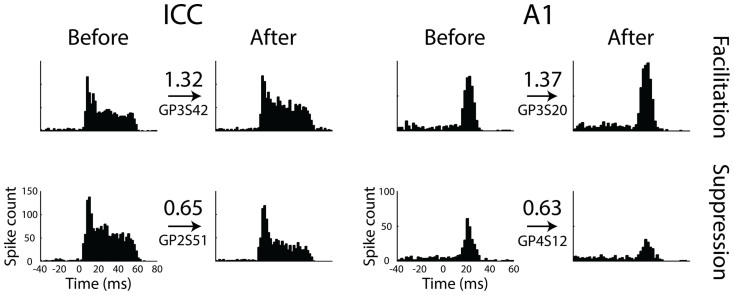
Representative examples of the effect of the stimulation paradigms. Peri-stimulus time histograms (1 ms bins) of multi-unit activity are plotted in response to 100 trials of a 50 ms duration, 70 dB SPL broadband noise stimulation presented before and after a 4,000 trial stimulation paradigm. The left and right columns are ICC and A1 responses, respectively, and the top and bottom rows are examples of significantly facilitated and suppressed sites, respectively. The abscissa time values are relative to the onset of acoustic stimulation, the numerical values above the arrows represent the change in acoustic-driven spike count (After) relative to the response for the baseline (Before) condition, and the GP and S numbers represent the animal and site number, respectively, for each example. The ICC and A1 facilitation examples are a result of the mSync-AE paradigm, the ICC suppression example is a result of the mSync-EA paradigm, and the A1 suppression example is a result of the Electrical Only paradigm.

**Figure 3:**
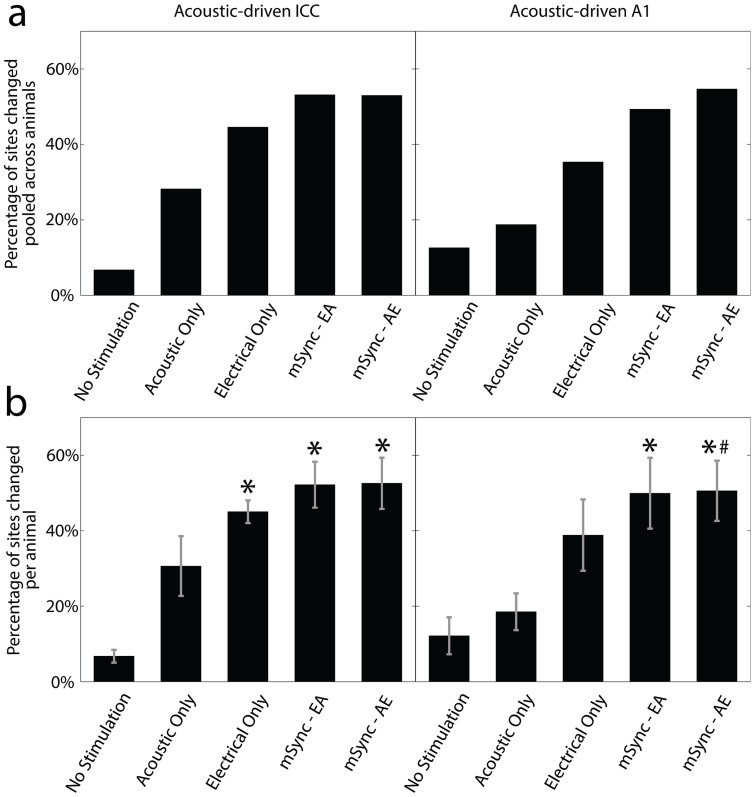
Percentage of sites in ICC and A1 with significantly changed acoustic-driven responses from different stimulation paradigms in Experiment 1. (a) The percentage of significantly changed sites (includes suppressed and facilitated sites) for each of the five stimulation paradigms are shown for the ICC (left panel) and A1 (right panel). Percentages are relative to the total number of sites (specified as n below) in the corresponding brain region that is pooled across all animals (specified as m below) for the given stimulation paradigm. ICC: No Stimulation (n = 192, m = 6), Acoustic Only (n = 241, m = 12), Electrical Only (n = 249, m = 12), mSync-EA (n = 239, m = 12), mSync-AE (n = 219, m = 12); A1: No Stimulation (n = 214, m = 9), Acoustic Only (n = 256, m = 9), Electrical Only (n = 300, m = 12), mSync-EA (n = 300, m = 12), mSync-AE (n = 300, m = 12). (b) The average percentages across animals for the given stimulation paradigm are shown in the ICC (left panel) and A1 (right panel). Error bars represent the standard error for visualization purposes. Asterisks (*) indicate percentages that are significantly different than the No Stimulation paradigm and the pound (#) symbol signifies the one that is significantly different than the Acoustic Only paradigm. All pairwise comparisons were performed using an unequal variance two-tailed t-test on ranked data (i.e., percentages) across animals with a criteria of p < 0.05 with a Bonferroni correction for multiple comparisons. The same n and m values from (a) apply to (b) in which the animals had an average site number and standard deviation in the ICC of 22.5 ± 6.6 and in A1 of 25.4 ± 6.1.

The five stimulation paradigms had various effects on acoustic-driven firing in the central auditory system. While some recording sites in the ICC and A1 exhibited no changes in firing rates, Figure 2 shows examples of sites that have been significantly facilitated (top panels) or suppressed (bottom panels) as a result of one of the stimulation paradigms. Overall, mSync paradigms induced a higher percentage of sites that were significantly changed than the control paradigms in both the ICC (Figure 3a; left panel) and A1 (Figure 3a; right panel). The No Stimulation paradigm affected the fewest sites overall for both the ICC and A1 as expected, though there were still some sites that exhibited significant changes in activity (ICC: 6.8%; A1: 12.6%). These changes may be attributed to inherent fluctuations in firing rates, physiological alterations associated with electrode implantation, or changes in anesthetic depth. The Acoustic Only paradigm resulted in the next fewest sites with significant changes in activity (ICC: 28.2%; A1: 18.8%). These results may partially explain why acoustic stimulation therapies for tinnitus have shown some success in suppressing the phantom percept^25^,^26^, possibly by altering central auditory neurons. The Electrical Only paradigm affected more sites (ICC: 44.6%, A1: 35.3%) than the Acoustic Only or No Stimulation paradigms, which is interesting in that stimulation of non-auditory pathways could induce greater plasticity within the central auditory system compared to direct auditory activation with an acoustic stimulus. Encouraging for the objective of this study, both mSync paradigms resulted in the greatest percentage of sites in the ICC and A1 with significant changes in spiking activity (mSync-EA – ICC: 53.1%, A1: 49.3%; mSync-AE – ICC: 53.0%, A1: 54.7%) when compared to the three control paradigms.

In addition to comparing the percentages of significantly changed sites pooled across all animals for the different stimulation paradigms, we were able to compare the average percentages across animals for each stimulation group and perform statistical analysis (Figure 3b). The average percentages per animal are quite similar to the percentages pooled across all animals. There is some variability across animals, which may be attributed to intrinsic differences among animals, different recording locations within the ICC and A1, and ordering effects of different stimulation paradigms (i.e., different cumulative effects from preceding paradigms since we randomized paradigms across animals). We cannot isolate these different factors with our current data set and protocol. The rationale for performing the experiments with this protocol along with several steps for further characterization of these different factors in future experiments are explained in the Methods. Nevertheless, Figure 3b shows that both mSync paradigms caused a significantly higher percentage of changed sites than the No Stimulation paradigm. In the ICC, the Electrical Only paradigm also showed a significantly higher percentage compared to the No Stimulation paradigm. In A1, mSync-AE showed a significantly higher percentage compared to the Acoustic Only paradigm. There were no other significant pairings in Figure 3b. Due to the small sample sizes for each stimulation group, we used an unequal variance two-tailed t-test on ranked data (i.e., percentages) across animals with a criteria of p < 0.05 that was adjusted with a Bonferroni correction for 10 pairwise comparisons^24^.

These data confirm that the mSync paradigms are not only causing a higher percentage of sites that exhibit significant changes in activity in the ICC and A1 compared to the control paradigms (Figure 3a), but also inducing a significantly higher percentage of changed sites compared to the No Stimulation paradigm that does not consistently occur for the other control paradigms.

### Experiment 1: Stimulus-timing dependent plasticity

**Figure 4:**
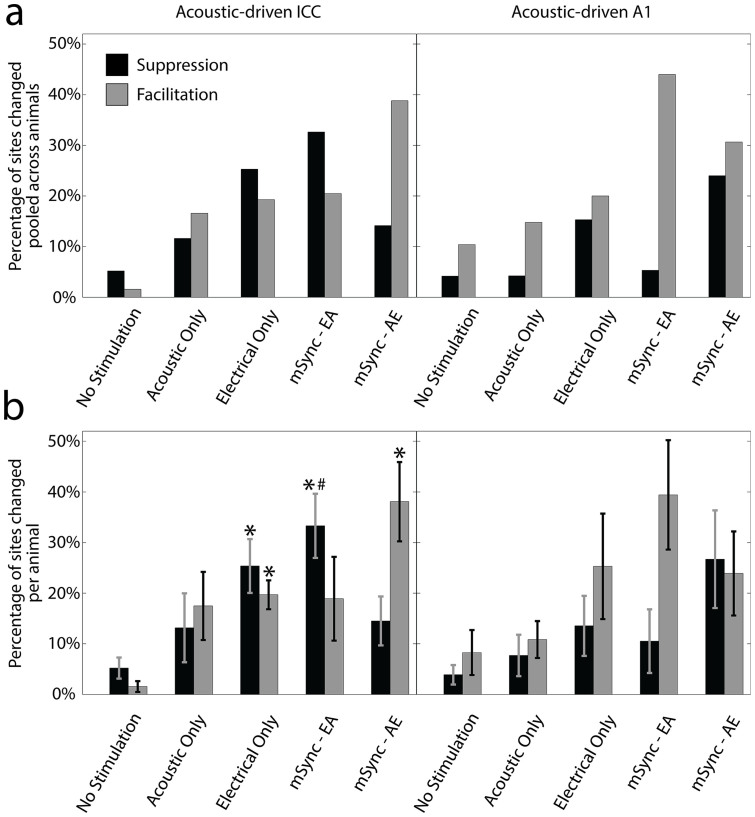
Suppression and facilitation of acoustic-driven responses in ICC and A1 for different stimulation paradigms in Experiment 1. The data from Figure 3 are separated into suppression (black bars) and facilitation (gray bars) in terms of total percentages pooled across animals (a) and average percentages across animals (b). The n and m values, average site numbers and standard deviations, and statistical analyses are the same as in Figure 3. Asterisks (*) indicate percentages that are significantly different than the No Stimulation paradigm and the pound (#) symbol signifies the one that is significantly different than the Acoustic Only paradigm.

While the mSync paradigms produced the most overall changes in the ICC and A1, we ultimately were interested in whether we could control the type of modulation by varying parameters such as interstimulus interval. We separated the pooled percentages of significant changes into suppression and facilitation for the ICC and A1, which are plotted in Figure 4a. Interestingly, the two mSync paradigms resulted in very different effects. In the ICC, the mSync-EA paradigm resulted in more sites being suppressed than facilitated while the mSync-AE paradigm resulted in more sites being facilitated than suppressed. The sole difference between these two stimulation paradigms was the relative timing of the stimulation, with mSync-EA consisting of electrical body stimulation presented 5 ms before the acoustic stimulation and mSync-AE consisting of the reverse stimulation order. Therefore, it appears that this stimulation paradigm is inducing a form of stimulus-timing dependent plasticity (**STDP**) in which one delay reinforces or facilitates neural firing while the opposite delay produces suppression of firing^27^.

In Figure 4b we also plotted the average percentages across animals for the different stimulation paradigms and performed the same statistical analysis for the suppression and facilitation groups as in Figure 3b. Consistent with Figure 4a, the left panel of Figure 4b shows that within the ICC, mSync-EA but not mSync-AE caused significantly more sites to be suppressed than the No Stimulation and Acoustic Only paradigms whereas mSync-AE but not mSync-EA caused significantly more sites to be facilitated than the No Stimulation paradigm. Electrical Only caused significantly more sites that were both suppressed and facilitated than the No Stimulation paradigm. These results demonstrate that even though the Electrical Only paradigm can induce both suppressive and facilitatory changes in the ICC, the advantage of using mSync is that the extent of suppression versus facilitation can be controlled by varying the interstimulus interval.

**Figure 5:**
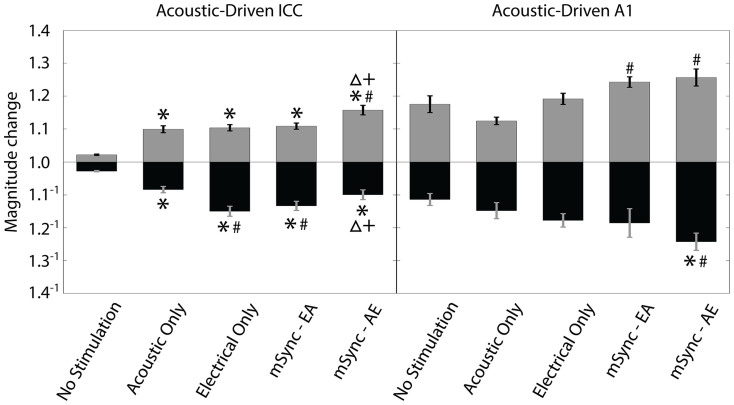
Magnitude change of acoustic-driven spike counts within ICC and A1 caused by different stimulation paradigms in Experiment 1. The magnitude change is calculated as the spike count for 100 trials presented immediately after the given stimulation paradigm relative to the baseline spike count. The magnitude changes were separated into sites that were suppressed (black bars) and facilitated (gray bars) for each stimulation paradigm regardless of whether the changes were significant or not. Error bars represent the standard error for visualization purposes. A one-way ANOVA followed by a Bonferroni-adjusted t statistic multiple comparison test was performed on the data with a criteria of p < 0.05. Asterisks (*) indicate distributions that are significantly different than the No Stimulation paradigm, the pound symbols (#) signify those that are significantly different than the Acoustic Only paradigm, the triangles (Δ) correspond to those that are significantly different than the Electrical Only paradigm, and the pluses (+) correspond to those that are significantly different than the mSync-EA paradigm. The n and m values and average site numbers and standard deviations are the same as in Figure 3.

The magnitude changes of ICC firing immediately after a stimulation paradigm relative to baseline for the five stimulation paradigms are shown in the left panel of Figure 5, and are generally consistent with what is expected based on Figure 4. We pooled the data across animals because we did not observe any significant trends on an animal-by-animal basis within each stimulation group performing a one-way ANOVA followed by a Bonferroni-adjusted t statistic multiple comparison test on the data with a criteria of p < 0.05. This consistency in results across animals matches what we also observed in Figures 3 and 4. The pooled magnitudes were then compared across different stimulation paradigms using a one-way ANOVA followed by a Bonferroni-adjusted t statistic multiple comparison test on the data with a criteria of p < 0.05. For all sites that were suppressed to any degree (black bars), the magnitudes for the mSync-EA and Electrical Only paradigms are suppressed to a significantly stronger degree than those for the No Stimulation, Acoustic Only and mSync-AE paradigms. For all sites that were facilitated (gray bars), mSync-AE magnitudes are significantly higher than the magnitudes for all of the other conditions. All stimulation paradigms had significantly higher magnitudes for suppression or facilitation compared to that of the No Stimulation paradigm. These results are consistent with those in Figure 4 and indicate that the Acoustic Only and Electrical Only paradigms can significantly modify neural activity in the ICC; however, by using paired stimulation with mSync, different interstimulus intervals can be implemented to induce varying or greater extents of suppression versus facilitation.

Similarly in A1, the two mSync paradigms produced different effects on neural activity. mSync-EA resulted in much more facilitation than suppression, which is the opposite of the effects shown in the ICC, while mSync-AE produced more equal amounts of suppression and facilitation (Figure 4; right panels). As shown in the right panel of Figure 5, mSync-AE was the only paradigm that exhibited significantly stronger suppression of neural activity for suppressed sites than that of both the No Stimulation and Acoustic Only paradigms. For the facilitatory sites, both mSync paradigms exhibited significantly higher magnitude changes than that of the Acoustic Only paradigm. Although there were only a small percentage of A1 sites that exhibited significant changes in activity for the No Stimulation paradigm (right panels of Figure 4), we were surprised to see such large magnitude changes for the No Stimulation paradigm (right panel of Figure 5) considering that there was no actual stimulation for these cases. It is not clear what may be causing these changes, though similar reasons as those described in the previous section could have occurred during the experiments. Similar to the ICC results, altering interstimulus intervals for mSync resulted in differential effects, though the transmission times may not have been optimized in A1 to get significant and opposing changes in plasticity (i.e., relating to STDP) as occurred within the ICC, which is further discussed in the Discussion.

### Experiment 2

In Experiment 1, we confirmed that mSync modulates neural activity in the ICC and A1 that depends on the interstimulus interval between acoustic and somatosensory stimulation. These results were achieved by stimulating four different body locations, paired with broadband noise, for 4,000 total trials. The goals of the second set of experiments (7 animals) were to determine if mSync could modulate central auditory neurons using fewer trials and to determine the effect of stimulating just one body location at a time for each mSync paradigm. For this set of experiments, we repeated a similar protocol as in Experiment 1 except that each mSync paradigm consisted of only 1,000 trials of electrical stimulation (at 2 trials/s) which was performed on only one body region at a time paired with broadband noise. We used just one interstimulus interval (mSync-EA) for all of the paradigms. We used additional body locations from Experiment 1 that included the tongue, neck, right mastoid, left mastoid, right shoulder, left shoulder, and back (Figure 1b). Two control paradigms (Acoustic Only and No Stimulation, 1000 trials each; Figure 1e) were also performed.

### Experiment 2: Single site mSync paradigms alter firing of ICC and A1

**Figure 6:**
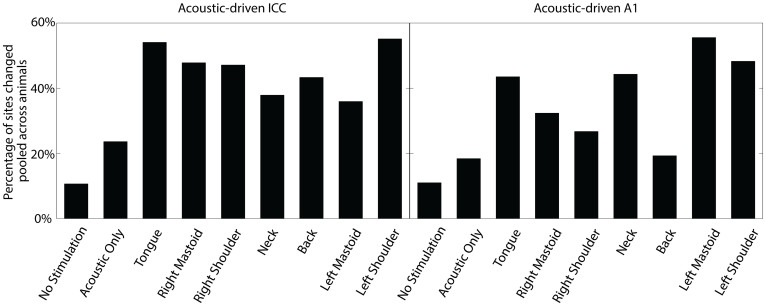
Percentage of sites in ICC and A1 with significantly changed acoustic-driven responses from different stimulation paradigms in Experiment 2. The percentage of significantly changed sites (includes suppressed and facilitated sites) for the two control paradigms and the seven body locations are shown for the ICC (left panel) and A1 (right panel). Percentages are relative to the total number of sites (specified as n below) in the corresponding brain region that is pooled across all animals (specified as m below) for the given stimulation paradigm. ICC: No Stimulation (n = 122, m = 6), Acoustic Only (n = 55, m = 3), Tongue (n = 135, m = 6), Right Mastoid (n = 115, m = 6), Right Shoulder (n = 104, m = 5), Neck (n = 132, m = 6), Back (n = 90, m = 4), Left Mastoid (n = 128, m = 6), Left Shoulder (n = 136, m = 6); A1: No Stimulation (n = 118, m = 5), Acoustic Only (n = 114, m = 4), Tongue (n = 108, m = 5), Right Mastoid (n = 62, m = 3), Right Shoulder (n = 86, m = 3), Neck (n = 79, m = 5), Back (n = 57, m = 2), Left Mastoid (n = 90, m = 4), Left Shoulder (n = 58, m = 3). The animals had an average site number and standard deviation in the ICC of 22.0 ± 5.5 and in A1 of 23.5 ± 9.6.

The nine stimulation paradigms (seven mSync and two control paradigms) all yielded significant changes in acoustic-driven activity in the ICC and A1. Overall, each of the mSync paradigms caused a higher percentage of sites to be significantly changed in the ICC and A1 than the control paradigms (Figure 6), and within the range of percentages observed in Experiment 1 even though much fewer trials were used in Experiment 2 (1000 trials instead of 4000 trials). Future studies can investigate the minimum number of trials of mSync to induce sufficient auditory plasticity that will be important for human treatment. From Figure 6, it can be seen that there was some variability in the percentage of sites that were significantly altered in the ICC and A1 depending on the stimulated body location. For instance, stimulation of the tongue and left shoulder produced the highest percentage of changes in the ICC, and relatively fewer sites in A1 were changed by stimulation of the back.

**Figure 7:**
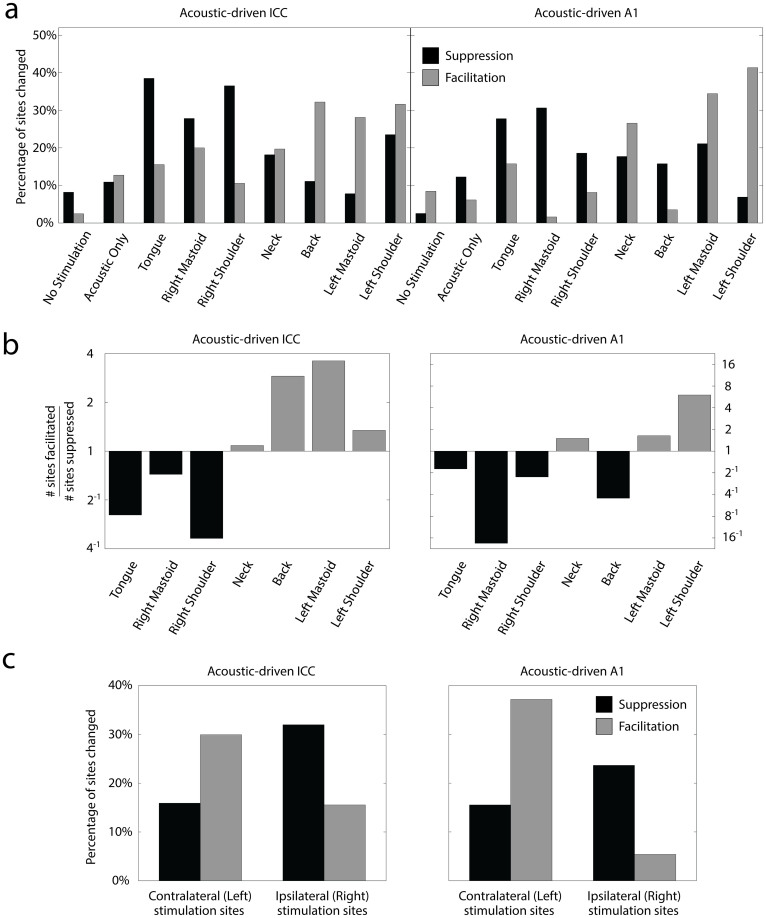
Suppression and facilitation of acoustic-driven responses in ICC and A1 for different stimulation paradigms in Experiment 2. (a) The total percentages pooled across animals from Figure 6 are separated into suppression (black bars) and facilitation (gray bars). The n and m values and average site numbers and standard deviations are the same as in Figure 6. (b) The experimental data from (a) is replotted as the number of sites significantly facilitated divided by the number of sites significantly suppressed for each body location. Bars in black are predominantly suppressive and bars in gray are predominantly facilitatory. (c) Sites in the ICC or A1 from (a) were pooled together according to the side of the body (shoulders and mastoids) stimulated with mSync for those sites. The percentage of sites that were significantly suppressed (or facilitated) by mSync with a left body site is shown in black (or gray) on the left half of each plot labeled as “Contralateral (Left) stimulation sites”. The percentage of total sites that were suppressed (or facilitated) by mSync with a right body site is shown in black (or gray) on the right half of each plot labeled as “Ipsilateral (Right) stimulation sites”. Site totals (n) were derived by assuming independence across animals (labeled m below) and stimulation protocols. ICC: Left (n = 264, m = 6), Right (n = 219, m = 6); A1: Left (n = 148, m = 4), Right (n = 148, m = 4).

**Figure 8:**
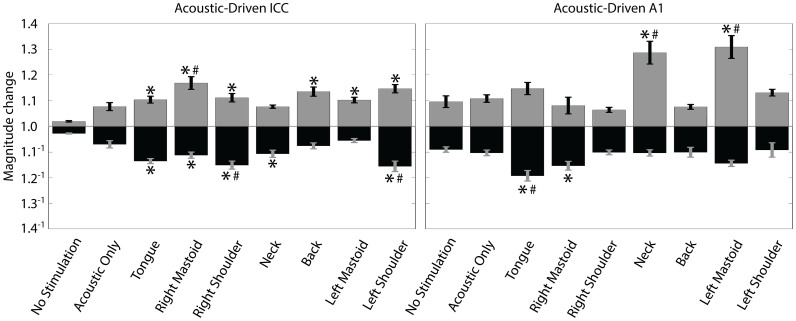
Magnitude change of acoustic-driven spike counts within ICC and A1 caused by different stimulation paradigms in Experiment 2. The magnitude change is calculated as the spike count for 100 trials presented immediately after the given stimulation paradigm relative to the baseline spike count. The magnitude changes were separated into sites that were suppressed (black bars) and facilitated (gray bars) for each stimulation paradigm regardless of whether the changes were significant or not. Error bars represent the standard error for visualization purposes. A one-way ANOVA followed by a Bonferroni-adjusted t statistic multiple comparison test was performed on the data with a criteria of p < 0.05. For clarity, only the significant pairings relative to the control paradigms are shown. Asterisks (*) indicate distributions that are significantly different than the No Stimulation paradigm and the pound symbols (#) signify those that are significantly different than the Acoustic Only paradigm. The n and m values and average site numbers and standard deviations are the same as in Figure 6.

For Experiment 2, it was not possible to perform statistical analysis on the average percentages across animals for each stimulation group as in Figures 3b and 4b because we had fewer animals per condition. As explained in the Methods, we minimized the total number of animals for this study even with the large number of stimulation paradigms by using 32-site electrode arrays to record from numerous sites per animal and by presenting several different stimulation paradigms to each animal. The number of recording sites for Experiment 2 was sufficient to show overall trends in total percentage values, as in Experiment 1, while also obtaining statistical differences in the magnitude changes across stimulation paradigms, as presented later in the results. Based on these initial encouraging results, including those presented in Figures 7 and 8, we can pursue future studies to assess the plasticity effects of specific stimulation paradigms in a greater number of animals as well as monitor the time course of these neural changes in the brain.

### Experiment 2: Effect of body location on neuromodulatory effects

Separating the overall changes into suppressive versus facilitatory effects provides greater insight into the effect of stimulating different body locations. Figure 7a shows the percentage of sites that were significantly suppressed or facilitated in response to each stimulation paradigm. There appears to be high variability across body locations, but the trends are more clearly evident when plotting the numbers of sites changed as a ratio of facilitated sites to suppressed sites as shown in Figure 7b. Stimulation of the tongue and right body sites suppressed firing to a greater extent in the ICC and A1, while stimulation of the neck and left body sites facilitated firing to a greater extent in the ICC and A1. Stimulation of the back exhibited mixed results, facilitating more responses in the ICC but suppressing more responses in A1. It is interesting that stimulation of left and right body locations caused different effects, with locations on the left causing greater facilitatory responses in the contralateral ICC and A1 (i.e., we recorded neural activity in the right ICC and A1) and locations on the right causing greater suppressive responses in the ipsilateral ICC and A1 (Figure 7c).

The magnitude changes of ICC and A1 firing after a stimulation paradigm relative to baseline for the stimulation paradigms are shown in Figure 8. The magnitudes were compared using a one-way ANOVA followed by a Bonferroni-adjusted t statistic multiple comparison test on the data with a criteria of p < 0.05. We found several of the body locations to be significantly higher or lower than the control paradigms. However, the magnitude changes in Figure 8 did not fully match the trends observed for the percentages shown in Figure 7. In other words, the body locations (e.g., tongue, right mastoid, right shoulder) that exhibited a greater percentage of suppressive versus facilitatory sites did not always exhibit larger suppressive versus facilitatory changes compared to the control paradigms, and vice versa. In the ICC, all seven mSync paradigms induced significantly larger magnitude changes than one or both of the control paradigms. In A1, four of the seven mSync paradigms induced significantly larger magnitude changes than one or both of the control paradigms. The Acoustic Only paradigm did not significantly differ in magnitude changes compared to the No Stimulation paradigm. In summary, mSync with a given body location can induce significant suppressive or facilitatory changes on a higher percentage of sites within the ICC and/or A1 compared to the control paradigms, but this does not mean that those altered sites will necessarily have larger suppressive or facilitatory changes in magnitude, respectively, and vice versa. Furthermore, mSync can induce significantly larger magnitude changes compared to the control paradigms by combining it with specific body locations.

## Discussion

Our mSync approach using paired broadband noise and somatosensory stimulation is capable of inducing significant neurophysiological changes in deep brain and cortical regions. The mSync paradigms produced a higher percentage of sites with significant changes in acoustic-driven firing in the ICC and A1 than control paradigms. Furthermore, switching the relative timing of the two modalities of stimulation or the location of body stimulation (e.g., the lateralization of sites) caused vastly different effects on neural activity within the ICC and A1, demonstrating the ability to control neuromodulatory effects by varying stimulus parameters. mSync using multiple pathways provides a potentially noninvasive way to systematically modulate and steer plasticity within pathogenic neural populations that may be relevant for various brain disorders.

Multimodal integration of auditory and somatosensory inputs occurs in several locations in the brain. Within the central auditory system, the dorsal cochlear nucleus (**DCN**) receives somatosensory input from both the dorsal column and trigeminal brainstem nuclei, while auditory input is received from the ventral cochlear nucleus and auditory nerve^28^,^29^,^30^. Bimodal stimulation of auditory and somatosensory inputs has previously been shown to alter coding properties in DCN pyramidal cells^28^,^31^,^32^, which then project to the IC^33^. Within the midbrain, the external IC (**ICX**) responds to both somatosensory and auditory inputs^30^,^34^ and stimulation of ICX has been shown to alter neural activity in the ICC^35^,^36^. At higher levels of the central auditory system, both the auditory thalamus and cortex are also altered by converging somatosensory and auditory inputs^32^,^37^,^38^. Outside of the traditional auditory pathway, one established multimodal integration center is the superior colliculus^39^, which has reciprocal projections with the IC^40^,^41^. Any combination of these pathways may be implicated in contributing to the neural changes shown in this study, as each of the auditory processing centers also have direct and indirect reciprocal projections to each other^30^.

The interstimulus timing effects observed in Experiment 1, at least in the ICC, seem to be a form of Hebbian STDP^27^, in which greater suppressive effects were observed for a delay in one direction (mSync-EA) but greater facilitatory effects were observed for a delay in an opposite direction (mSync-AE). These findings are consistent with previous studies that invasively stimulated the spinal trigeminal nucleus (a somatosensory brainstem region) paired with pure tone stimulation and demonstrated interstimulus timing effects within the DCN and A1^32^,^42^ that may be relevant for tinnitus and its treatment^43^. In addition, STDP has been shown in the DCN within slice preparations^44^ and in A1 of anesthetized and awake ferrets using stimulation with pure tones of different frequencies^45^. It is possible that we did not clearly observe STDP in A1 due to misalignment in transmission times for converging activation of neurons in response to acoustic and somatosensory stimulation. The interstimulus intervals used for mSync may have been ideal for showing a reversal in neural effects in the ICC but were not optimized for A1. It is also possible that the timing differences were not optimized for all neurons within the nuclei, as we did not have complete suppressive or facilitatory effects in the ICC and A1. Numerous studies have shown different types of neurons in the ICC and A1 have a wide range of acoustic-driven latencies^46^,^47^, and it is also expected these neurons would have different latencies to somatosensory inputs. Therefore, further research is needed to confirm that these varying transmission times were the cause of the different results in the ICC versus A1, as well as for individual neurons sampled within each region. If true, this would open up the possibility for more accurately targeting different neurons by adjusting the interstimulus interval for mSync and inducing differential effects appropriate for a given brain disorder.

The results of Experiment 2 indicate that mSync utilizing ipsilateral body locations relative to the recorded ICC and A1 generally suppresses acoustic-driven firing, while stimulation of contralateral body locations generally facilitates firing. This is indirectly supported by previous studies which have indicated that suppression of the DCN could be induced via ipsilateral somatosensory stimulation^48^,^49^. This result is significant as both tinnitus and hyperacusis have been linked to hyperactivity across the central auditory system^19^,^20^,^21^,^23^. Therefore, it would be logical to attempt to suppress this hyperactivity by using mSync with body locations ipsilateral to the hyperactive brain regions. It is important to note that we assessed changes in acoustic-driven patterns within the central auditory system in response to mSync. However, other abnormal patterns in spontaneous activity, synchrony, and temporal firing have also been linked to tinnitus and possibly for hyperacusis^23^. It will be interesting to investigate the effects of mSync on these other neural patterns, especially in disease-specific animal models, in future studies.

Stimulation of the tongue for mSync also had drastic effects on neural activity along the central auditory pathway, including suppressing the largest percentage of sites in the ICC and the second highest percentage of sites in A1. Previous studies have demonstrated that the tongue can be effectively used to modulate activity in the central nervous system^50^,^51^. Tongue stimulation has been used as a sensory substitute for balance-impaired or blind subjects. For instance, repeated electrical stimulation of the tongue induced plasticity in the balance-processing network and improved behavioral measures in individuals with balance dysfunction that could be sustained for weeks^52^,^53^. Tongue stimulation was also recently shown to improve gait in patients with chronic multiple sclerosis^54^. The question remains if mSync using tongue stimulation combined with precisely timed activation of other body sites and additional modalities, such as sound and visual stimuli, can provide further improvements in treatment.

From a clinical perspective, one immense challenge as well as opportunity in translating mSync to patients is the vast number of parameters that can be explored, including but not limited to the pathways being activated (auditory, visual, somatosensory, motor, cognitive, limbic), type of stimulation (acoustic, electric, magnetic, pressure, thermal, ultrasound), stimulation level, interstimulus interval, and stimulus waveform. The key importance of this large parameter space is that stimulation could be tailored specifically to each patient, which would begin to address the issue of inter-patient variability. In addition, it is possible that patients would be able to individually navigate through different interactions of these parameters outside of the clinic to identify optimal settings that would fix or suppress specific neurons driving their abnormal brain network. It is encouraging that this initial proof-of-concept study activating only two pathways with somewhat arbitrary parameters was able to induce significant and systematic neural changes in both deep brain and cortical structures. Based on the success of this preliminary study, future work will seek to determine appropriate parameters and multimodal pathways for inducing neural plasticity across different auditory and non-auditory centers and for targeting specific neurons that are involved with a given brain disorder. Additionally, future studies will need to address how cumulative effects from repeated stimulation and with specific sequences of stimulation paradigms alter activity in the brain.

This study demonstrates that mSync can induce controlled plasticity within deep brain and cortical structures by varying stimulus parameters. In order for mSync to become a viable therapeutic option for clinicians, several questions still need to be addressed. Can mSync locally target and alter specific aberrant neural populations that are relevant for a given neurological disorder while not significantly altering other populations, as predicted by the four assumptions described in the Introduction? While short-term plasticity was revealed in this study, can mSync induce long-lasting effects to allow for a one-time or periodic treatment protocol? And what is the long-term safety and efficacy of such a treatment? Despite these questions, the mSync concept opens up a new approach and opportunity for expanding the use of neuromodulation to a larger patient population in need of a reliable and noninvasive therapeutic option.

## Methods

### Animal surgery and electrode implantation

Basic surgical procedures are similar to those presented in previous studies^55^,^56^. Experiments were performed on 20 young Hartley guinea pigs (315–430 g; Elm Hill Breeding Labs, Chelmsford, MA) within an acoustically- and electrically-shielded chamber and using Tucker-Davis Technology hardware (Alachua, FL) and custom Matlab software (Natick, MA). These experiments were performed in accordance with policies and protocols approved by the University of Minnesota Institutional Animal Care and Use Committee. Each animal was anesthetized with an intramuscular injection of a mixture of ketamine (40 mg/kg) and xylazine (10 mg/kg) with 0.1 mL supplements every 45–60 minutes to maintain an areflexive state. Atropine sulfate (0.05 mg/kg) was injected into the neck muscle periodically to reduce mucous secretions in the airway. Heart rate and blood oxygenation were continuously monitored via a pulse oximeter and body temperature was maintained at 38.0 ± 0.5°C using a heating blanket and rectal thermometer.

A craniotomy was performed to expose the right visual and auditory cortices, and two 32-site electrode arrays (NeuroNexus Technologies, Ann Arbor, MI) were inserted into the right ICC and A1 (Figure 1a). The ICC array consists of four 8 mm long shanks separated by 500 μm with eight iridium sites linearly spaced 200 μm along each shank (site area = 703 µm^2^). The array was inserted through the occipital cortex into the ICC at a 45° angle off the sagittal plane to align it with the tonotopic gradient. The cortical array consists of four 5 mm long shanks separated by 500 μm with eight iridium sites linearly spaced 200 μm along each shank (site area = 413 µm^2^). The array was placed perpendicular to the cortical surface and inserted to a depth of approximately 1.6 mm. ICC and A1 site impedances typically ranged between 0.8-3.0 MΩ. The A1 recording ground wire was implanted into the brain near the intersection of the midline and bregma and the ICC ground was positioned in the neck muscle. After the probes were confirmed to be in the correct location, the brain was covered with agarose to reduce swelling, pulsations, and drying during the recording sessions.

### Placement of recording and stimulation electrodes

Acoustic-driven neural responses were used to verify the placement of our electrodes within the ICC and A1. Acoustic stimulation was presented to the animal's left ear canal via a speaker coupled to a custom-made hollow ear bar. The speaker-ear bar system was calibrated using a 0.25 in condenser microphone (ACO Pacific, Belmont, CA). Multi-unit neural data was sampled at a rate of 25 kHz, passed through analog DC-blocking and anti-aliasing filters up to 7.5 kHz, and digitally filtered between 0.3 and 3.0 kHz for analysis of neural spikes. Spikes were determined as voltages exceeding 3.5 times the standard deviation of the noise floor.

To create frequency response maps (**FRM**s), pure tones (60 ms duration, 0.5 ms rise/fall time) of varying frequencies (0.625–40 kHz, 8 steps/octave) and levels (0–70 dB SPL in 10 dB steps) were randomly presented (4 trials/parameter), and the acoustic-driven spike rates were calculated for responses recorded in the ICC (taken 5–60 ms after tone onset) and A1 (5–20 ms after tone onset). Best frequencies (**BF**s) were calculated from the FRMs as the frequency centroid at 10 dB above the visually determined threshold. Array placements within the ICC were confirmed by observing sharply-tuned FRMs that systematically increased in BF with increasing depth^56^,^57^. FRMs for sites outside of the ICC in external regions of the inferior colliculus typically exhibit broad and weak tuning and/or multiple FRM peaks and were excluded from the analysis in this paper. A1 was identified as the region lateral of the pseudosylvian sulcus that exhibited increasing BFs in the rostrolateral-to-caudomedial direction and short response latencies of approximately 12–20 ms based on previous studies^58^,^59^. ICC recording sites were pooled across animals and frequency regions for analysis. A1 recording sites were pooled across animals, frequency regions, and cortical layers for analysis.

Subcutaneous needle electrodes were placed onto or within the tongue, neck, right mastoid, left mastoid, right shoulder, left shoulder, or back (Figure 1b) for somatosensory stimulation. The tongue electrode was placed on top of the tongue and extended fully into the mouth, taking care not to puncture the tongue, and fixed in place by the mouthpiece of the stereotaxic frame. The neck electrode was placed along the spine of the animal halfway between the ears and the shoulder joints. The mastoid electrodes were positioned along the mastoid bone groove. The shoulder electrodes were placed at the shoulder joints, and the back electrode was placed along the spine halfway down the animal's back. The stimulation ground was distributed across four subcutaneous needle electrodes placed into the animal's four limbs. For this initial study, we used subcutaneous needles rather than surface electrodes for stimulating the somatosensory pathways since they were easier to position in a consistent way and could achieve activation with much lower currents by avoiding the high impedance interface of the animal's skin and fur. Future studies will make use of noninvasive electrodes or pressure or thermal actuators to activate the somatosensory pathways.

### General experimental protocol

Different stimulation paradigms, including mSync and control paradigms, were presented to the animals as described in greater detail within the experimental protocols below and depicted in Figure 1c-e. For spike analysis, we compared the acoustic-driven activity in response to broadband noise (50 ms duration, 0.5 ms rise/fall time, 70 dB SPL, equal energy between 625 Hz and 40 kHz) played immediately before and after a particular stimulation paradigm. Spike counts were measured over a 50 ms window for ICC responses and a 30 ms window for A1 responses starting at the onset of the acoustic-driven response. A change in acoustic-driven activity was determined as significant using an unequal variance two-tailed t-test on ranked data (i.e., spike counts) across trials with a criteria of p < 0.01^24^. When comparing significant differences in modulatory effects among the various stimulation paradigms, statistical tests with Bonferroni corrections were performed that are specifically described in the Results.

### Experiment 1: Interstimulus delay

In 13 animals, we investigated whether mSync could modulate acoustic-driven responses in a stronger and more systematic manner than control paradigms and whether different interstimulus delays would lead to different types of neural modulation. Since we did not initially know which locations would be effective in inducing auditory plasticity and based on the clinical cases in modulating tinnitus with manipulations of the head or neck described in the Introduction^17^,^18^, we selected the neck, tongue, left mastoid, and right mastoid for mSync stimulation. In unpublished preliminary studies, we also observed that stimulation of these four body regions typically resulted in low thresholds of activation within the IC and AC.

We performed five stimulation paradigms of 4,000 trials each (2 trials/s, ~33 minutes for each paradigm): No Stimulation (control), Acoustic Only (control), Electrical Only (control), mSync-EA (electrical body stimulation preceded acoustic stimulation by 5 ms), and mSync-AE (acoustic stimulation preceded electrical body stimulation by 5 ms), as depicted in Figure 1d. Acoustic stimulation consisted of a 50 ms duration, 50 dB SPL broadband noise (equal energy between 625 Hz and 40 kHz) and electrical stimulation consisted of three biphasic, charge-balanced, cathodic-leading pulses (205 μs/phase) presented at 200 Hz. Electrical body stimulation levels were set as high as possible without inducing any noticeable motor response, varying between 0.22–0.63 mA across experiments. This criterion was based on established acupuncture protocols in which needles are inserted through the skin and electric current is applied to them^60^. Using current levels below 1 mA and twitching threshold, electroacupuncture generally activates the large somatosensory touch fibers rather than the smaller diameter fibers associated with pain and temperature that can require current levels 5–10 times larger. For the Electrical Only, mSync-EA, and mSync-AE paradigms, each of the four body locations was stimulated one at a time (for all three pulses) for 1,000 trials in a randomized order, resulting in a total of 4,000 trials. We used this somatosensory stimulation protocol because we did not have sufficient time per experiment to look at the effects of both interstimulus delay and specific body locations. Thus, Experiment 1 focuses on the effects of interstimulus delay while Experiment 2 focuses on the effects of specific body locations.

The five stimulation paradigms were presented in a random order across animals with an hour between each paradigm to reduce cumulative effects. Based on unpublished preliminary studies, these different stimulation paradigms can induce changes in auditory activity that can last beyond several hours, and thus we realize that one hour between paradigms may not be sufficient to completely avoid cumulative effects. The challenge with the types of experiments performed in this study is that there are a large number of stimulation parameters that need to be tested, the corresponding neural changes can last for varying periods of time across parameters, and the types of changes can depend on which neurons are being recorded within the ICC or A1. For this initial study, we focused on testing a reasonable number of stimulation paradigms through Experiments 1 and 2. In order to minimize the number of animals required for this study while still having a large number of recording sites for each stimulation paradigm, we used 32-site arrays placed in the ICC and A1 for each animal and also reduced the time between each stimulation paradigm to one hour to present several conditions per animal. If we observe significant trends in the data, even with the confounding factors described above, then we know that some trends exist and can perform future studies to more systematically assess the plasticity effects over time and for different locations within the ICC and A1. We can also assess the ordering effects of different stimulation paradigms, which was not possible in this study due to the randomization used in the stimulation protocol that was further confounded by the different recording locations within the ICC and A1 across animals.

Acoustic-driven activity in the ICC and A1 was recorded in response to 100 trials of 70 dB SPL broadband noise (2 trials/s) immediately before and after each paradigm to compare the changes in neural activity caused by a given stimulation paradigm. While tinnitus or hyperacusis may be linked to other response patterns, such as hyperactivity or hypersynchrony of spontaneous activity and changes in temporal firing^19^,^20^,^21^,^23^, we initially focused on acoustic-driven responses since they can be reliably recorded and compared for the different paradigms in an anesthetized preparation and using multi-unit recordings, and this answers our main question as to the ability of mSync to induce controllable auditory plasticity. Spontaneous activity was generally low or nonexistent in our anesthetized preparation, especially in A1, and single-unit recordings required for synchrony and temporal firing analysis was not readily possible when using multi-site arrays. Future studies will therefore investigate these other neural features in an awake preparation using single-unit recordings, especially in disease-specific animal models.

### Experiment 2: Body location

After finding convincing neuromodulatory effects in Experiment 1, we investigated the effects of using fewer trials of stimulation and specific body locations in 7 animals. Three stimulation paradigms were used in this study: No Stimulation (control), Acoustic Only (control), and mSync (Figure 1e). For mSync paradigms, we limited the interstimulus delay to mSync-EA (electrical body stimulation preceded acoustic stimulation by 5 ms). Subcutaneous needle electrodes in these experiment were placed onto or within the tongue, neck, right mastoid, left mastoid, right shoulder, left shoulder, and back. Each body location was investigated separately, as opposed to the protocol in Experiment 1. Also, stimulation was performed for only 1,000 trials (2 trials/s, ~8 minutes of stimulation). Electrical body stimulation levels ranged between 0.32–0.63 mA across these experiments. As in Experiment 1, acoustic-driven activity in the ICC and A1 was recorded in response to 100 trials of 70 dB SPL broadband noise (2 trials/s) immediately before and after each paradigm to compare the changes in neural activity caused by a given stimulation paradigm. The nine stimulation paradigms were presented in a random order across animals with an hour between each paradigm to reduce cumulative effects.

## Author Contributions

C.D.M., B.T.S., C.D.G. and H.H.L. conceived and designed the study, C.D.M. and B.T.S. performed the experiments and analyzed the data, C.D.M. and H.H.L. wrote the initial manuscript, and C.D.M., B.T.S., C.D.G. and H.H.L. provided input on the study and approved the final manuscript.
